# Creation of a functional hyperthermostable designer cellulosome

**DOI:** 10.1186/s13068-019-1386-y

**Published:** 2019-02-28

**Authors:** Amaranta Kahn, Sarah Moraïs, Anastasia P. Galanopoulou, Daehwan Chung, Nicholas S. Sarai, Neal Hengge, Dimitris G. Hatzinikolaou, Michael E. Himmel, Yannick J. Bomble, Edward A. Bayer

**Affiliations:** 1 Department of Biomolecular Sciences, The Weizmann Institute of Science, 7610001 Rehovot, Israel; 2 Faculty of Natural Sciences, Ben-Gurion University of the Negev, 8499000 Beer-Sheva, Israel; 3 Microbiology Group, Faculty of Biology, National and Kapodistrian University of Athens, Zografou Campus, 15784 Athens, Greece; 4 Biosciences Center, National Renewable Energy Laboratory, Golden, CO USA; 5 Present Address: Division of Chemistry and Chemical Engineering, California Institute of Technology, Pasadena, CA 91125 USA

**Keywords:** Multi-enzyme complex, Cellulases, Thermostability, *Caldicellulosiruptor bescii*, Scaffoldin, Dockerin, Cohesin

## Abstract

**Background:**

Renewable energy has become a field of high interest over the past decade, and production of biofuels from cellulosic substrates has a particularly high potential as an alternative source of energy. Industrial deconstruction of biomass, however, is an onerous, exothermic process, the cost of which could be decreased significantly by use of hyperthermophilic enzymes. An efficient way of breaking down cellulosic substrates can also be achieved by highly efficient enzymatic complexes called cellulosomes. The modular architecture of these multi-enzyme complexes results in substrate targeting and proximity-based synergy among the resident enzymes. However, cellulosomes have not been observed in hyperthermophilic bacteria.

**Results:**

Here, we report the design and function of a novel hyperthermostable “designer cellulosome” system, which is stable and active at 75 °C. Enzymes from *Caldicellulosiruptor bescii*, a highly cellulolytic hyperthermophilic anaerobic bacterium, were selected and successfully converted to the cellulosomal mode by grafting onto them divergent dockerin modules that can be inserted in a precise manner into a thermostable chimaeric scaffoldin by virtue of their matching cohesins. Three pairs of cohesins and dockerins, selected from thermophilic microbes, were examined for their stability at extreme temperatures and were determined stable at 75 °C for at least 72 h. The resultant hyperthermostable cellulosome complex exhibited the highest levels of enzymatic activity on microcrystalline cellulose at 75 °C, compared to those of previously reported designer cellulosome systems and the native cellulosome from *Clostridium thermocellum*.

**Conclusion:**

The functional hyperthermophilic platform fulfills the appropriate physico-chemical properties required for exothermic processes. This system can thus be adapted for other types of thermostable enzyme systems and could serve as a basis for a variety of cellulolytic and non-cellulolytic industrial objectives at high temperatures.
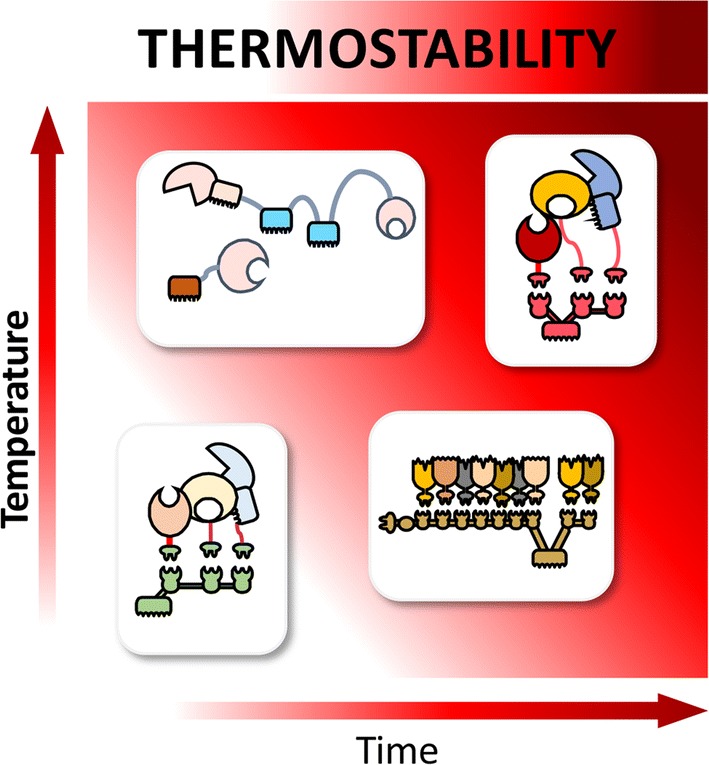

**Electronic supplementary material:**

The online version of this article (10.1186/s13068-019-1386-y) contains supplementary material, which is available to authorized users.

## Background

During the past decade, renewable energy has gained mounting interest, and extensive research has been dedicated to overcome the techno-economic barriers that continue to prevent its implementation into energy systems of all scales [1]. Cellulosic biomass is the most abundant source of renewable energy on earth. Its deconstruction into soluble sugars *en route* to biofuel production would allow the conversion of both waste and dedicated crops into energy [2–5]. Notwithstanding ongoing efforts that have been employed to convert cellulosic waste into soluble sugars, the cost of such a process is still not competitive with the use of fossil-derived energy. The main obstacle in this context stems from the high recalcitrance of lignocellulosic substrates [6] and cellulose in particular.

Deconstruction of cellulose is carried out by complementary enzymes: i.e., (i) endoglucanases that randomly cleave cellulose chains internally, (ii) exoglucanases that cleave either the exposed reducing or non-reducing extremities of the chain end into cellobiose, and (iii) β-glucosidases that cleave the cellobiose product into two molecules of glucose. A subgroup of endoglucanases is referred to as processive endoglucanases, which have been shown to sequentially hydrolyze cellulose chains internally, but continue to cleave the cellulose chain in a processive fashion [7–10]. Yet, the use and production of the various cellulases remain costly, due to problematic production steps and demanding process parameters, such as optimizing concentrations, pH, and maintenance of ambient temperatures throughout an exothermic process [11]. In this context, thermostable cellulolytic enzymes are particularly attractive candidates for biomass deconstruction. Their resistance and robustness to high temperatures can allow faster and more effective reactions as well as extended enzyme survival following harsh chemical pre-treatment conditions [12]. In fact, owing to the elevated reaction temperatures, pre-treatment conditions may be relieved or even eliminated in biomass-to-biofuels conversion processes [13].

Cellulases are either secreted as free enzymes or integrated into multi-enzymatic complexes called cellulosomes. In the cellulosome, the enzymes act in high synergy while being targeted, directly and in collective fashion, to the substrate [14–17]. Cellulosomes exhibit specific modular architectures, composed of a non-catalytic “scaffoldin” platform, which contains multiple cohesin modules for integration of the various enzymes, through their dockerin modules, and a carbohydrate-binding module (CBM) for targeting the intact enzyme-laden complex to the substrate [18].

Designer cellulosomes are artificially self-assembled chimaeric protein complexes, which can be used as a tool for comparative study of cellulose degradation and can also serve to improve cellulose deconstruction [19–23]. Designer cellulosomes are self-assembled from chimaeric cellulosomal components: i.e., chimaeric cohesin-containing scaffoldin(s) and chimaeric dockerin-bearing enzymes [24]. The chimaeric scaffoldin consists of a CBM module that allows targeting to the substrate and several cohesin modules of divergent species with different specificities. The chimaeric enzymes possess complementary and specific dockerin modules attached to their catalytic component. The designer cellulosome thus enables control of the number, composition and positioning of the selected enzymes and their integration into a given chimaeric scaffoldin.

Cellulosomes have been described in anaerobic, mainly mesophilic, bacteria [25], except for isolated species of the genus *Clostridium* that possesses some bacteria that grow at relatively high temperatures (from 50 to 65 °C) [15, 25, 26]. More recently, other mildly thermophilic cellulosome-producing bacteria have been classified in the genera *Herbinix* and *Herbivorax* [27–29]. Nevertheless, to date, no cellulosomal systems have been reported in hyperthermophilic bacteria.

Mesophilic and some thermophilic free enzymes have been successfully converted into cellulosomal enzymes by grafting therein a dockerin module. However, the functionality and stability of the resultant designer cellulosomes were limited to temperatures of up to 60 °C and no higher [30–34]. In the present work, we examined whether hyperthermophilic free enzymes could be integrated into designer cellulosomes and whether the resultant complexes would remain stable and functional at high/extreme temperatures. For this purpose, the enzymes of the genus *Caldicellulosiruptor w*ould be particularly attractive candidates for integration into designer cellulosomes.

*Caldicellulosiruptor bescii* has been described as the most thermophilic bacterium capable of growing on crystalline cellulose and other cellulosic and lignin-containing substrates [35–37]. The bacterium produces free cellulolytic enzymes, with optimal activities up to temperatures of 85 °C [12, 38–46]. The genome of this bacterium has been sequenced [47] and encodes for many multi-modular cellulase proteins that contain multiple CBMs and catalytic modules (CAZy DSM 6725 http://www.cazy.org/b890.html). In fact, it has long been known that the genus *Caldicellulosiruptor* (née *Anaerocellum* and *Caldocellum*) produces such multifunctional enzymes [35, 48–52]. These enzymes could thus be considered collectively as an intermediate strategy between the free and cellulosomal system; indeed, both substrate channeling between the catalytic modules and the targeting of the catalytic units occurs in multifunctional enzymes and designer cellulosomes [53].

In the present work, we integrated hyperthermostable enzymatic components from *Ca. bescii* and specific thermophilic cohesin–dockerin modular pairs into designer cellulosomes to assess their functionality at extreme temperatures. For this purpose, we examined the functional thermal limits of the enzymatic complex using an endoglucanase as a model, and then assembled a complete trivalent designer cellulosome with complementary enzymatic functions. At 75 °C, the performance of the hyperthermostable designer cellulosome exceeded that of the native *Clostridium thermocellum* cellulosome, which was impaired at such high temperatures.

## Methods

### Cloning

The genomic DNA of *Ca. bescii* (DSM 6725) was used as a template for the cloning of endoglucanase Cel5D with and without its CBM28. GH5-*g*, GH5-*t* and GH5-*v* were cloned in pET28a (Novagen, Darmstadt, Germany) using the gDNA template, primers, and restriction enzymes listed in Additional file 1: Table S1. The dockerin sequences were determined like explain in Kahn et al. [54]. PCRs were performed with Phusion High Fidelity DNA polymerase F530-S (New England Biolabs, Inc, Massachusetts, United States), PCR products, and plasmids were synthetized with Fastdigest enzymes (Thermo scientific, USA). Ligation was performed with T4 DNA ligase (Fermentas UAB, Vilnius, Lithuania). PCR products were purified using a HiYield™ Gel/PCR Fragments extraction kit (RBC Real Biotech, Valencia, CA).

GH9-lk-*v*, GH9-*v*, GH48-*lk*-*t*, GH48-*t* were synthesized in pET21a by GenScript (USA). All enzymes were equipped with a His-Tag for purification by immobilized metal ion affinity chromatography (IMAC). The monovalent scaffoldins Scaf*T*, Scaf*G*, and Scaf*V* and the trivalent scaffoldin Scaf*GTV* were described previously [33, 55–57]. Competent *Escherichia coli* XL1 cells were used for plasmid maintenance and production.

### Protein expression and purification

All the proteins were expressed in BL21 (DE3) and BL21Star™ (DE3) competent cells; after reaching an A_600_ of ± 0.7–1 (2 h of growth at 37 °C) in 1 to 2 L Luria Broth (LB), supplemented with 2 mM CaCl_2_ and the appropriate antibiotic (50 mg/L of kanamycin or 100 mg/L of Ampicillin), bacterial cells were induced with 0.2 mM of isopropyl-1-thio-β-d-galactoside (IPTG) (Fermentas UAB Vilnius, Lithuania), and the culture was continued overnight at 16 °C. Cells were harvested by centrifugation at 4200*g* for 15 min. The pellets were re suspended in 30 mL TBS (Tris-buffered saline, 137 mM NaCl, 2.7 mM KCl, 25 mM Tris–HCl, pH 7.4) containing 5 mM imidazole. Cells were sonicated, and harvested at 22,000*g* for 30 min.

His-tagged proteins were purified on a Ni-nitriloacetic (NTA) column (Qiagen, Hilden, Germany) as described previously [58]. All scaffoldins were purified through macroporous bead cellulose pre-swollen gel (IONTOSORB, Usti nad Labem, Czech Republic) as described previously [34]. All purified proteins used for activity assays shown in Fig. 5 were subjected to a second purification step using a Superdex^®^ 26/60 200 PG column or a Superdex^®^ 10/300 75 GL. All proteins were assessed for purity according to their calculated molecular weight by SDS-PAGE and presence or absence of additional bands in the preparation. Concentration of the protein was measured by tryptophan absorbance at 280 nm, based on their extinction coefficient measured by the Protparam tool [58]. The proteins were subsequently stored in 50% (v/v) glycerol at − 20 °C, except for the proteins used for activity assays that were not supplemented with glycerol and stored at 4 °C. The *Thermotoga maritima* β-d-glucosidase was purchased from Megazyme (Bray, Ireland) and desalted using a Hi-trap 26/10 (GE life science) desalting column before use to remove the ammonium sulfate stabilizer.

Cellulosome samples were prepared from 3-day growth cultures of *Cl. thermocellum* ATCC 27405 by the affinity purification method as described by Morag et al. [59].

### Analysis of cohesin–dockerin interactions

The specific interaction of matching cohesin and dockerin modules was assayed by affinity-based ELISA as previously reported [60] by immobilizing (coating) one module (cohesin- or dockerin-containing fusion protein) onto wells of a microtiter plate and allowing the immobilized molecule to interact with its partner module (i.e., fusion protein counterpart, e.g., matching monovalent or trivalent cohesin-containing scaffoldin in the case of the immobilized dockerin-containing component, or matching enzyme-fused dockerins in the case of the immobilized cohesin-containing component). For each complex (i.e., enzyme and scaffoldin pair) the proper stoichiometric ratio was determined experimentally using non-denaturing PAGE: Samples (4 to 8 μg) of each protein at different molar ratios (from 0.4 to 1.6 enzyme:scaffoldin) were incubated at 37 °C for 1.5 to 2 h in 20 μL TBS, supplemented with 12 mM CaCl_2_ and 0.05% Tween 20 [61, 62]. Gels were run at 100 V (3% acrylamide stacking gel and 9% acrylamide separating gel).

### Thermostability assays

Each complex was formed as described in the cohesin–dockerin analysis of interaction section and assayed for thermostability. Samples were incubated in a thermocycler machine in as many equal volume aliquots at each time points. The aliquots were then separated in two fractions and run on SDS and non-denaturing PAGE as described previously [32, 62]. The band intensity was quantified using ImageJ freeware [63], and the relative band intensity was then calculated.

### Enzymatic assays

The optimal concentration for enzymes to be used in the linear range was first determined on carboxymethyl cellulose (CMC, Sigma-Aldrich, St Louis, MI, USA) at 1.5% w/v (final concentration). Samples were incubated at 75 °C for 10 min at various concentrations (from 0.02 to 0.5 μM). The 0.05 μM enzyme concentration was further used to compare all of the free enzymes as well as the single enzymes complexed to their monovalent scaffoldin activity on CMC (after 1.5 h incubation at 37 °C for complex formation). To terminate the reaction, samples were placed on ice.

The hydrolytic performance of equimolar amounts of the trivalent designer cellulosome complexes, as well as monovalent designer cellulosomes, the free enzymes, the wild-type enzymes and the purified native *Cl. thermocellum* cellulosome, were assayed on microcrystalline cellulose (Avicel, Sigma-Aldrich, St. Louis, Missouri, United States) as substrate (4% final concentration). Prior to substrate addition, each scaffoldin was incubated with equimolar quantities of the matching enzyme(s) (for a final concentration of 0.05 μM per protein) for 2 h at 37 °C in interaction buffer (TBS with added 10 mM CaCl_2_ and 0.05% Tween 20). The activity was carried out for 24 h to 72 h at 60 °C and 75 °C in a Lumitron (Petah-Tikva, Israel) shaker incubator at 900 rpm in 50 mM final concentration of acetate buffer, pH 5.5. Enzymatic reactions were terminated by placement of the reaction tube on ice. Tubes were then centrifuged for 2 min at 17,000*g* at room temperature.

Soluble sugar production was evaluated as follows: 100 μL of each sample was supplemented with 150 μL of dinitrosalicylic acid (DNS) and then boiled for 10 min, as described previously (Miller GL). Dinitrosalicylic acid reagent was used for determination of reducing sugar. Absorbance were measured at 540 nm, and glucose was used a standard to determine released sugar concentration. Assays were performed two to three times in triplicate or duplicate, respectively. Scaf20L was complexed to three *Cl. thermocellum* enzymes in equimolar quantities, and in the same quantities as Scaf*GTV* and its three *Ca. bescii* enzymes. The activity was tested at 60 °C (optimal temperature of *Cl. thermocellum* cell cultures) and at 75 °C (i.e., the highest temperature at which designer cellulosomes are reportedly stable) [33].

The protein concentration of the *Cl. thermocellum* cellulosome used in Fig. 5 was calculated to be equivalent (in g/L) to that of the designer cellulosome.

The designer cellulosome used in Fig. 6 was produced and assembled as described in Stern et al. [34] paper.

## Results

### Library of hyperthermophilic cellulases and cellulosomal components

**Fig. 1:**
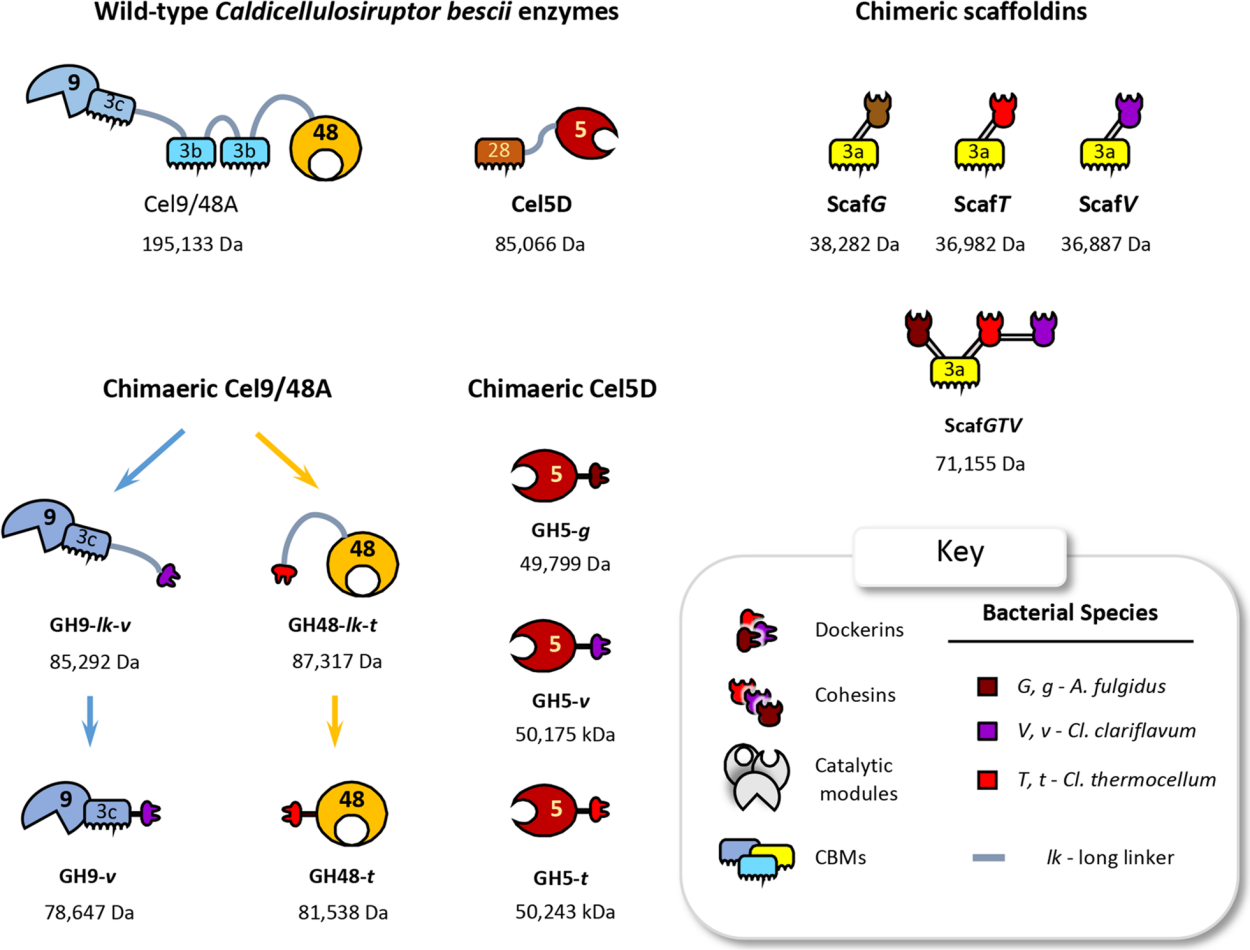
Schematic view of the proteins used in this study. The key defines the symbols used for the protein modules, which are assembled into a cellulosomal complex. The bacterial or archaeal source of each cohesin and dockerin module is color-coded as follows: red, *Cl. thermocellum*; purple, *Cl. clariflavum*; brown, *A. fulgidus*. Upper case characters (*T*, *V* and *G*) indicate the source of the cohesin modules and lower case (*t*, *v* and *g*) indicate the source of the dockerin module. All the catalytic modules originate from *Ca. bescii*, the numbers correspond to their GH family (GH9, GH48, GH5). A previously designed trivalent scaffoldin, Scaf*20L* [21], was also used as a control. In this case, two cohesins from mesophilic bacteria—*Acetivibrio cellulolyticus* (powder blue) and *Bacteroides cellulosolvens* (light green)—were used in addition to a cohesin from *Cl. thermocellum.* Scaf*20L* was used to incorporate orthologous *Cl. thermocellum* enzymes into a cellulosome to compare its action with the hyperthermophilic *Cl. bescii*-based complex prepared in this work

The wild-type and recombinant proteins used in this study are represented schematically in Fig. 1. The catalytic modules were selected from bifunctional cellulase Cel9/48A and endoglucanase Cel5D of known properties from the hyperthermophilic bacterium, *Ca. bescii*. The cohesins and dockerins were selected from components of thermophilic microbes, since mesophilic cohesin–dockerin interactions were shown to be unstable at high temperatures [33]. Thus, the same chimaeric thermostable scaffoldin (Scaf*GTV*) and thermostable dockerins (*g, t* and *v*) were used. The cohesin and dockerin components were derived from the thermophilic cellulosome-producing bacteria *Cl. thermocellum* (designated herein *T* and *t* in this species for the cohesin and dockerin, respectively) and *Cl. clariflavum* (*V* and *v*), and the hyperthermophilic archaeon, *Archaeoglobus fulgidus,* that also produces cohesin-/dockerin-containing proteins (*G* and *g*).

Cel9/48A, originally termed CelA in previous publications [51, 64] is a potent multifunctional cellulase from *Ca. bescii*, which contains a glycoside hydrolase family 9 (processive endoglucanase activity) and a family 48 (exoglucanase activity) catalytic domain, as well as three family 3 carbohydrate-binding modules (CBMs) connected via linker peptides [41, 65, 66]. This multi-modular cellulase is expressed constitutively and is the most abundant extracellular protein produced in *Ca. bescii.* Among all the *Ca. bescii* biomass-degrading enzymes, its cellulolytic activity plays the most prominent role in biomass degradation [43, 46, 66]. These properties led to its selection in the present work for integration into designer cellulosomes.

Cel9/48A possesses particularly long Pro/Thr rich linkers of identical sequence that likely play a role in the quaternary structure of the protein as well as in its enzymatic activity [40]. Various chimaeric constructs with these catalytic modules were therefore designed, with long (*lk*) or short linker segments in between the catalytic and the dockerin module. The two catalytic modules of Cel9/48A were separated, thus allowing better DNA manipulation as well as protein expression, purification and stability, prior to their integration into a designer cellulosome: i.e., each catalytic module was expressed separately, bearing a dockerin module grafted at their C-terminus, with or without a long linker. Thus, GH9-*lk*-*v* contained a 73-residue linker and GH48-*lk*-*t* a 64-residue linker with dockerins from *Cl. clariflavum* and *Cl. thermocellum,* respectively, whereas GH9-*v* and GH48-*t* were essentially without linkers (technically, they each had a very short linker of -3 and 7 amino acids, respectively). The GH9 chimeras were designed to selectively bind to a cohesin from *Cl. clariflavum* (*V*) and the GH48 chimeras to a cohesin from *Cl. thermocellum* (*T*).

In a previous work, the bifunctional *Ca. bescii* Cel9/48A enzyme was found to act in synergy with endoglucanase Cel5D from the same bacterium [67]. Cel5D was therefore selected to be integrated into our hyperthermophilic designer cellulosome. The Cel5D endoglucanase possesses a glycoside hydrolase 5 module and a CBM28 that has been found to bind to amorphous cellulose [68]. The CBM28 was removed and replaced by a dockerin, in order to convert the enzyme into the cellulosomal mode. The native enzyme also possesses three SLH domains at the C-terminus (for cell-wall anchoring) that were also removed in the present work. Since Cel5D is the simplest enzyme in our system, we used it to calibrate the functional limits of our hyperthermophilic system. The enzyme was thus converted to the cellulosomal mode by fusing the catalytic module (at the C-terminus) to three different dockerins derived from the three different thermophilic microbes mentioned above, thus generating GH5-*g*, GH5-*t*, and GH5-*v*.

The chimaeric scaffoldin Scaf*GTV* used in our hyperthermophilic designer cellulosome is a trivalent scaffoldin that contains three divergent thermophilic cohesins for binding enzymes that bear the matching dockerin. The CBM3a, derived from the native *Cl. thermocellum* scaffoldin, was also included in the chimaeric scaffoldin to allow binding to the cellulosic substrate [69]. This scaffoldin has been described and used in a previous study [33], and whereas the enzymes used previously could not function at temperatures higher than 60 °C, the scaffoldin was reported to be stable until 70 °C.

In the present work, monovalent scaffoldins, containing single cohesins, Scaf*G*, Scaf*T*, and Scaf*V*, were also used to examine the individual properties of the components of the trivalent scaffoldin under thermophilic and hyperthermophilic conditions. All monovalent scaffoldins contained the same CBM3a as Scaf*GTV*. In our experiments these scaffoldins served to test the thermostability of the three cohesins, to assay the thermostability of the cohesin–dockerin pairs when bound to an enzyme, to study the targeting effect of the CBM to the substrate and to determine the proximity effect of the enzymes within the trivalent scaffoldin.

Each protein was purified to apparent homogeneity, and exhibited the expected molecular masses as examined by 10 or 12% SDS-PAGE (Additional file 2: Figure S1).

### Functional binding activity of the dockerin-containing enzymes and scaffoldins

We demonstrated the specific binding of the individual cohesin–dockerin pairs, each of which exhibited species-specific interaction with negligible inter-species cross-reactivity, as demonstrated by ELISA (Additional file 3: Figure S2A). The binding of the different cohesins of the trivalent Scaf*GTV* was also examined, and the cohesins were shown to specifically recognize their matching dockerin (Additional file 3: Figure S2B). Furthermore, the stoichiometric molar ratio of each dockerin-bearing enzyme to its corresponding mono- or trivalent scaffoldin was determined by non-denaturing PAGE (Additional file 4: Figure S3), and the values obtained were used for further denaturation and activity experiments.

### The novel designer cellulosome components are stable at very high temperatures

**Fig. 2:**
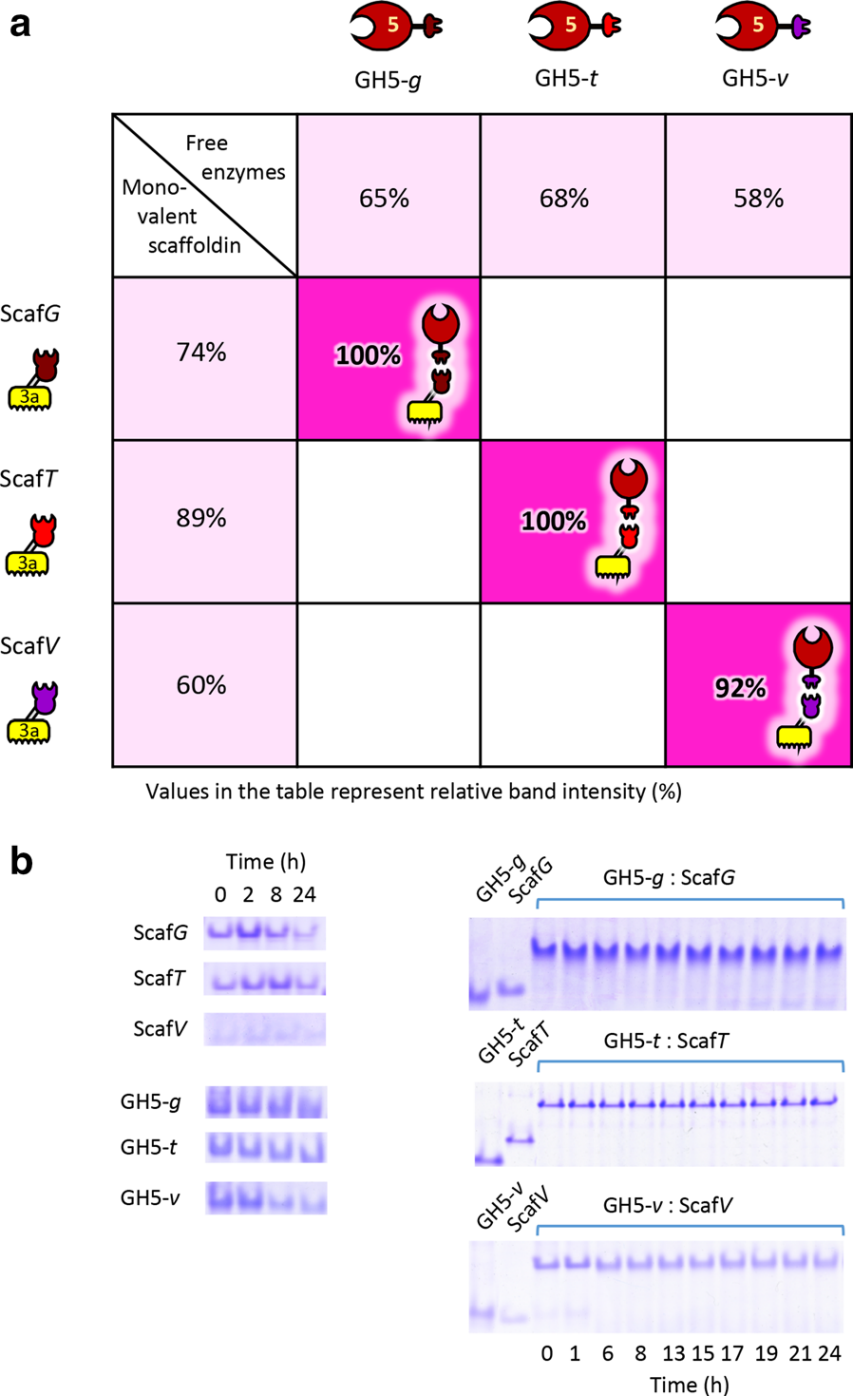
Thermostability of the monovalent scaffoldins (Scaf*G*, Scaf*T*, and Scaf*V*) and chimaeric enzymes (Ce5D-*g*, -*t*, -*v*) alone (pink) and in complex (magenta) determined by non-denaturing PAGE. **a** Relative band intensities following thermal treatment (75 °C, 24 h), quantified by ImageJ. **b** Non-denaturing PAGE documenting the relative thermostability of the various designer cellulosomes components, alone (left) or in complex (right), upon incubation at 75 °C for the indicated time intervals (between 0 to 24 h)

The enzymes GH5-*g*, GH5-*t*, GH5-*v,* attached to their corresponding monovalent scaffoldin counterparts (Scaf*G*, Scaf*T*, or Scaf*V*), were used to test the stability of the cohesin–dockerin pairs, incubated at 75 °C for 24 h, by assessing the comparative band intensities of the complex by non-denaturing PAGE (Fig. 2). Upon incubation, the free chimaeric enzymes retained 58% to 68% stabilities. Monovalent scaffoldins exhibited stabilities of 60% to 89%. Upon binding of the enzymes to the monovalent scaffoldins, stabilities of the complexes increased to 92% to 100%, suggesting that complex formation results in higher thermal stability of both the scaffoldin and enzyme components.

**Fig. 3:**
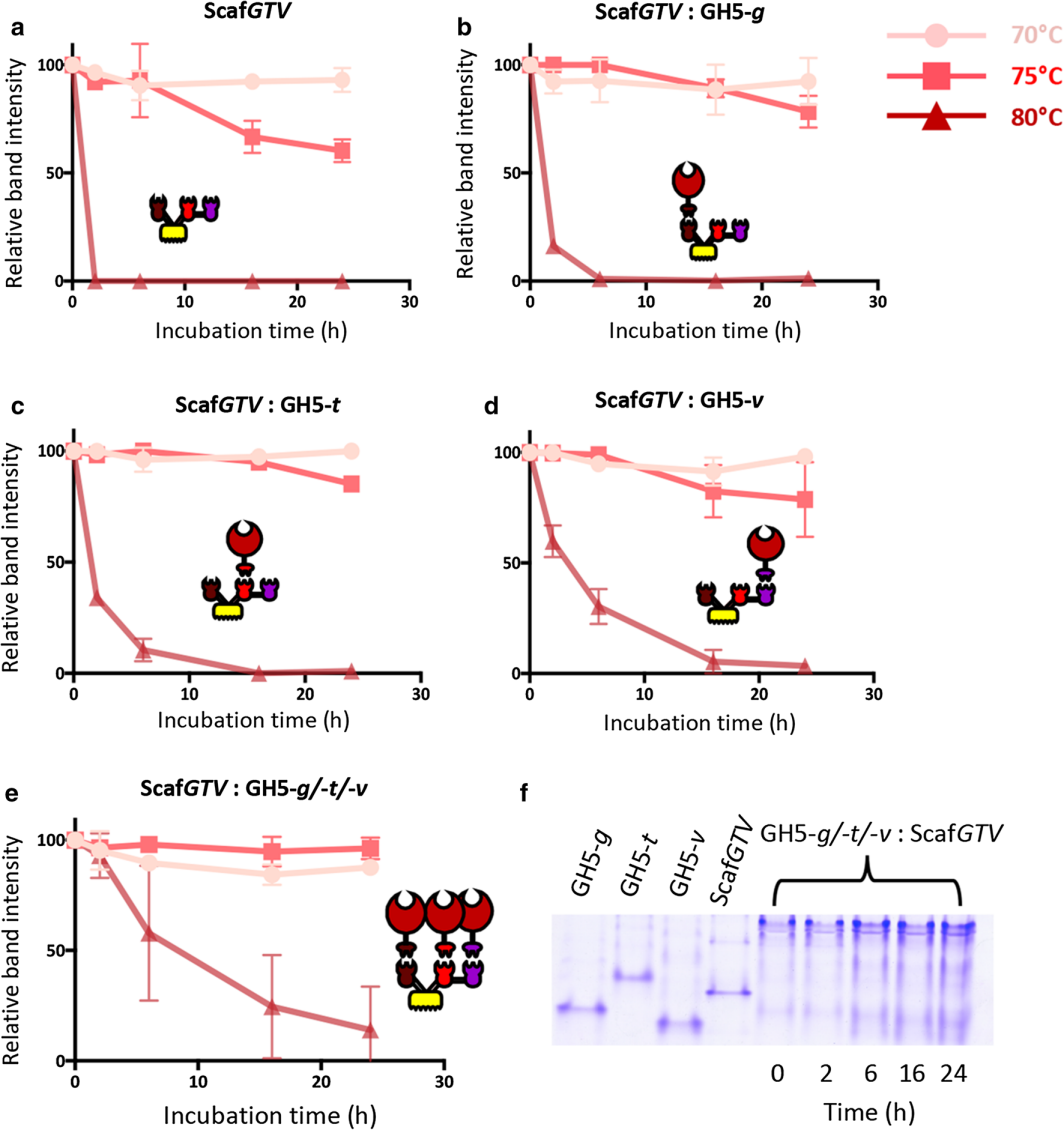
Thermostability of the chimaeric trivalent scaffoldin (Scaf*GTV*) either alone, or complexed to the different dockerin-bearing Cel5D chimeras. **a**–**e** Display the relative intensities of the designated bands on non-denaturing PAGE gels after 24 h of incubation at 70 °C, 75 °C and 80 °C, quantified by ImageJ. **f** An example of the corresponding gel bands of the three chimaeric dockerin-bearing Cel5G complexed to Scaf*GTV* incubated at 75 °C for 0 to 24 h. Denaturation assays were reproduced in triplicate, the data represent the mean ± SD

Dockerin-borne GH5 enzyme chimeras were used to test the stability of the trivalent Scaf*GTV* scaffoldin at high temperature (Fig. 3 and in Additional file 5: Figure S4). The Scaf*GTV* alone was ~ 100% and ~ 60% stable at 70 °C and 75°, respectively, after 24 h of incubation, while at 80 °C the protein was degraded within a few minutes (Fig. 3a and in Additional file 5: Figure S4A). Upon complexation of the trivalent scaffoldin, Scaf*GTV,* with each of the three chimaeric forms of Cel5D separately (Fig. 3b–d, and in Additional file 5: Figure S4B–D), the three different complexes showed complete stability at 70 °C. At 75 °C, the three complexes exhibited stability levels between 70 and 85%; and at 80 °C the complexes were 50% stable or less after a couple of hours incubation. The stability of the three enzymes, complexed on Scaf*GTV*, was examined for 24 h at the above temperatures (Fig. 3e and Additional file 5: Figure S4E): at 70 °C the full complex was ~ 100% stable and at 75 °C a slight decrease in stability was observed (96%). At 80 °C the tertiary complex was more stable than the single enzyme/scaffoldin complexes, since at that temperature the complex showed 50% degradation only after 6 h of incubation.

Furthermore, both Cel9 and Cel48 constructs (with and without linker) were tested under the same conditions (incubation for 24 h at 75 °C) with Scaf*GTV,* and the complexes exhibited full stability (data not shown). Taken together, our results indicate that our system is highly stable and can be further used and tested for enzymatic activity at 75 °C.

### The novel hyperthermophilic designer cellulosome degrades cellulose at high temperatures

Degradation of microcrystalline cellulose by the wild-type and chimaeric enzymes was examined in the free state (i.e., the enzymes in the absence of scaffoldin) or bound state (i.e., each enzymes is bound to its monovalent scaffoldin counterpart). All enzymes show activity in both states at 75 °C (Additional file 6: Figure S5). With the exception of GH48-*lk*-*t*, the enzymes bound to the monovalent scaffoldin exhibited higher levels of degradation of cellulosic substrates by releasing more soluble sugars than the free enzymes. Different architectures of designer cellulosome were further studied as described in the following sections.

### Effect of linker length between the dockerin and catalytic modules

We investigated the importance of the linker between the dockerin and catalytic modules, using chimeras with various linker lengths for *Ca. bescii* Cel9 and Cel48 enzymes. Interestingly, the GH9 enzyme with a long linker showed enhanced activity as opposed to the same enzyme with a short linker (Additional file 6: Figure S5). On the other hand, the GH48 enzyme with short linker showed no substantial difference in activity (1.1 fold) (Additional file 6: Figure S5).

**Fig. 4:**
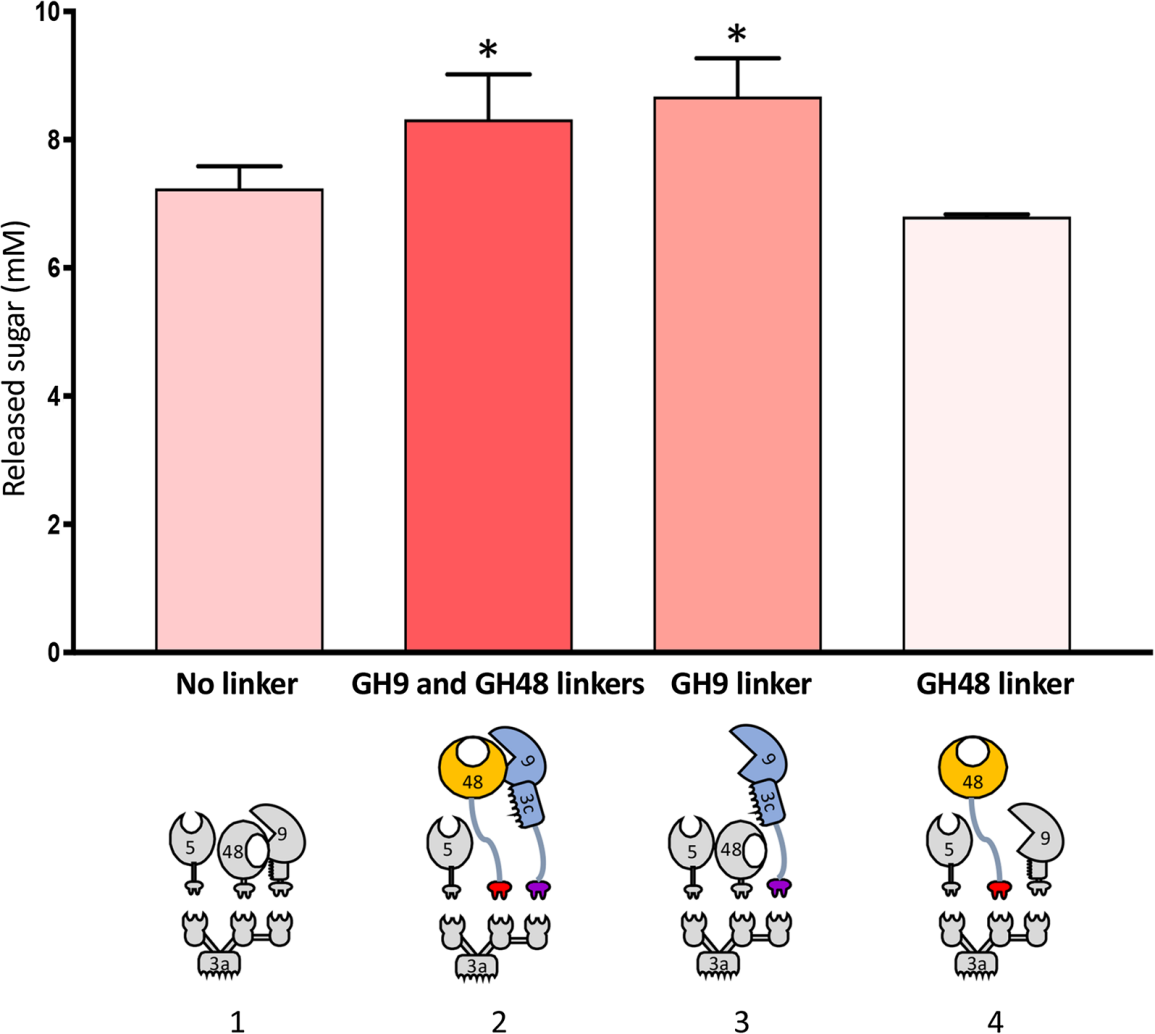
Effect of linker length in designer cellulosome enzyme components. Comparative degradation of microcrystalline cellulose by various forms of trivalent designer cellulosomes containing the following components: (1) the chimaeric GH5, GH9 and GH48 (with short linkers); (2) the chimaeric GH5, GH9 and GH48 (with linkers); (3) the chimaeric GH5, GH9 (with linker) and GH48 (without linker), and (4) the chimaeric GH5, GH9 (without linker) and GH48 (with linker) as indicated by the pictograms (as defined in Fig. 1). Enzymatic activity is defined as mM of released sugars after 24 h incubation of the enzymes with 4% of Avicel substrate at 75 °C. Each reaction was conducted in triplicate; the data represent the mean ± SD, where an asterisk (*) indicates *p *< 0.05 (two-tailed *t* test)

The trivalent Scaf*GTV* was thus complexed with the *Ca. bescii* GH5, GH9 and GH48 enzymes, with long or short linkers for GH9 and GH48, (all four combinations were tested, Fig. 4). After 24 h of assay, slightly higher activities were shown to occur using the long-linker GH9 together with either the short- or long-linker GH48. GH9 and GH48 with long linkers were chosen for subsequent designer cellulosome experiments assay, as they provided high activity and are closer to the native form of the parent bifunctional Cel9/48A enzyme.

### Comparison of the hyperthermophilic designer cellulosome to the free wild-type *Ca. bescii* enzyme system and to the wild-type *Cl. thermocellum* cellulosome

**Fig. 5:**
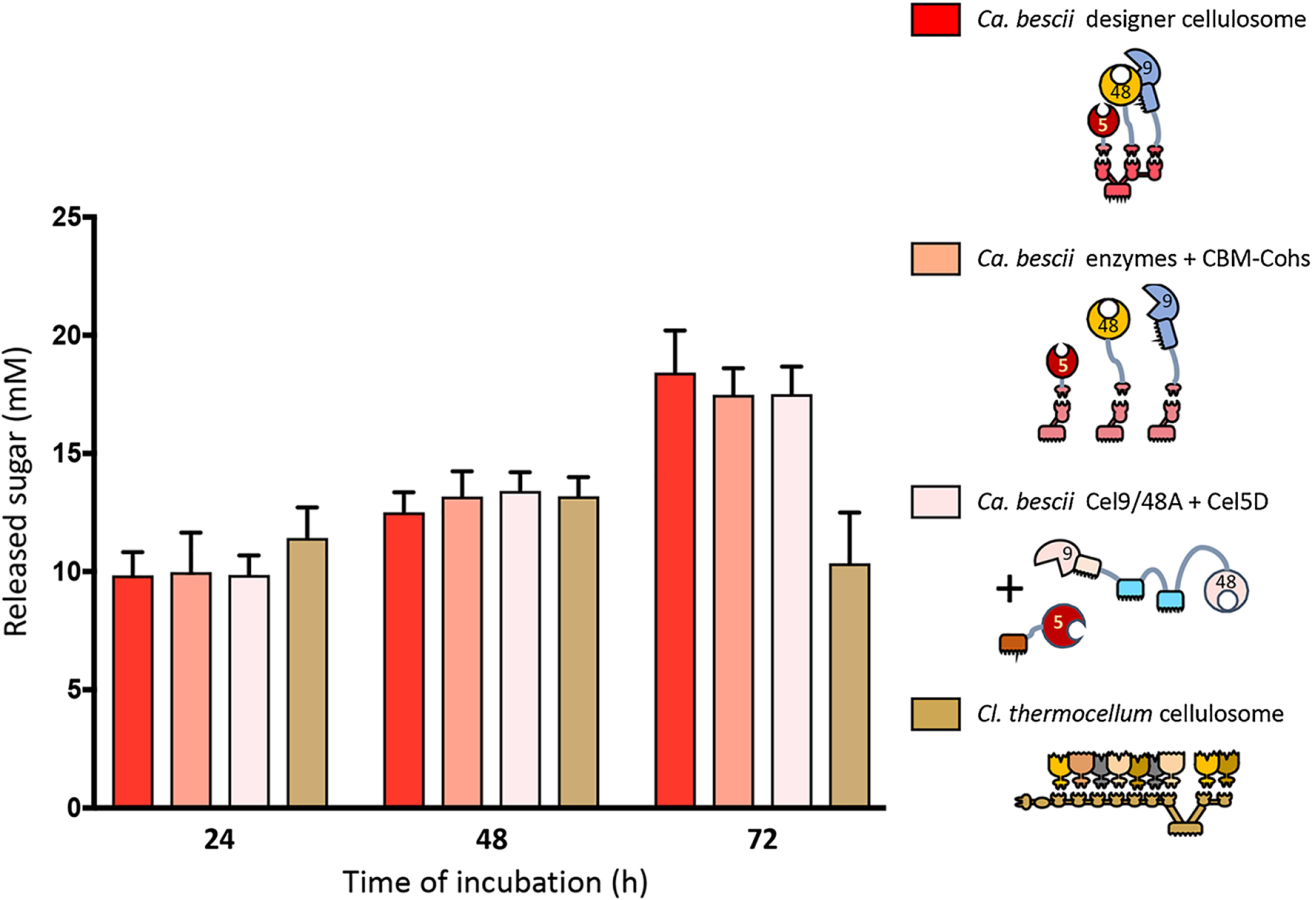
Comparison of the performance of the hyperthermophilic designer cellulosome to the wild-type systems. The relative activities of the *Ca. bescii*-based designer cellulosome (red bars), chimaeric *Ca. bescii* enzymes complexed to their monovalent scaffoldin (dark pink), wild-type uncomplexed *Ca. bescii* enzymes (light pink) and *Cl. thermocellum* cellulosome (tan) were determined at desired time intervals. Enzymatic activity is defined as mM of released sugars after 24 to 72 h incubation of the enzymes with 4% of Avicel substrate. Each reaction was conducted in triplicate; the data represent the mean ± SD

The cellulose-degrading performance of the resultant trivalent hyperthermophilic designer cellulosome was compared to the following enzyme combinations: (i) the same three enzymes complexed to their respective monovalent scaffoldins (thereby supplying the substrate-targeting function to the free enzyme system), (ii) the wild-type bifunctional *Ca. bescii* Cel9/48A enzyme produced in *E. coli* together with the *Ca. bescii* Cel5D endoglucanase (without the SLH module), and (iii) the native cellulosome of *Cl. thermocellum* (Fig. 5). All complexes were supplemented with a thermostable β-glucosidase, derived from *Thermotoga maritima* to prevent cellobiose feedback inhibition of the cellobiose product on susceptible enzymes. No significant differences were observed between the above-described systems after 24 and 48 h of reaction. However, after 72 h of activity at 75 °C, the three *Ca. bescii*-based systems showed an enhanced activity of 1.7–1.8-fold higher than that of the native cellulosome of *Cl. thermocellum*. These results indicate that the hyperthermophilic designer cellulosome complex is as active as the native system, and surpasses the native cellulosome of *Cl. thermocellum* at high temperatures after extended incubation periods.

### Comparison of the hyperthermophilic designer cellulosome to a thermostable designer cellulosome comprising *Cl. thermocellum* enzymes

**Fig. 6:**
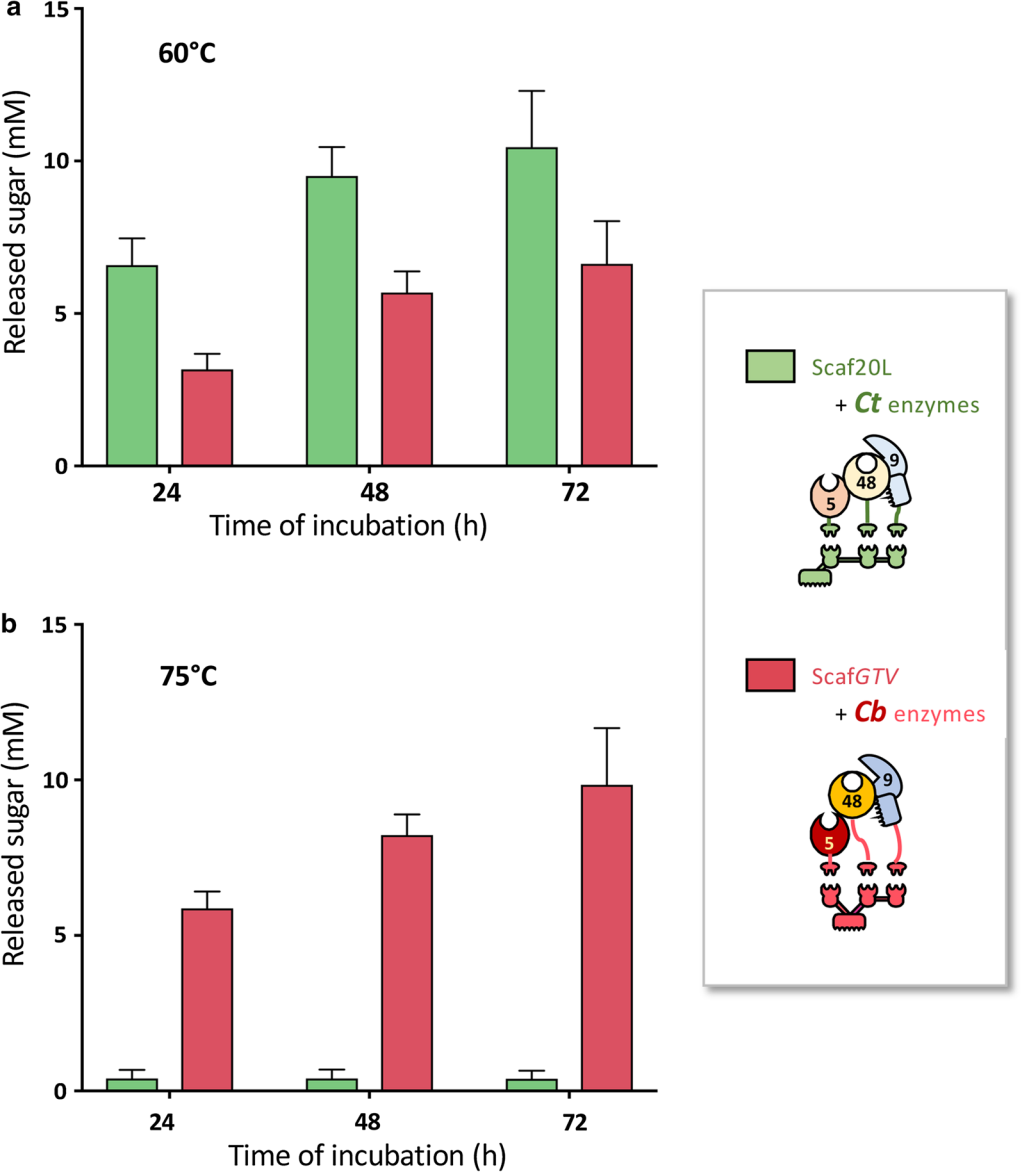
Comparison of the activity of *Cl. thermocellum*-based designer cellulosome (green bars) versus hyperthermophilic *Ca. bescii*-based designer cellulosome (red bars) at 60 °C (**a**) and 75 °C (**b**). The three enzymes of the two designer cellulosomes are from the same GH enzyme family: GH5, GH9 and GH48. Enzymatic activity is defined as mM of released sugars after 24 to 72 h incubation of the enzymes with 4% of Avicel substrate. Each reaction was conducted in triplicate; the data represent the mean ± SD

Three enzymes from the same GH family originating from *Cl. thermocellum* were reported to act synergistically in a designer cellulosome at 60 °C on a trivalent chimaeric scaffoldin, Scaf20L [34]. This designer cellulosome has an optimal temperature of 60 °C, and we compared its cellulose-degrading capacity to that of our hyperthermophilic designer cellulosome, at 60 °C (Fig. 6a) and at 75 °C (Fig. 6b). At 60 °C, the native *Cl. thermocellum* designer exhibited the highest activity, reaching levels 1.6- (72 h) to 2-fold (24 h) that of our hyperthermophilic designer cellulosome. However, at 75 °C, the *Cl. thermocellum*-based designer cellulosome showed only very low levels of activity (~ 0.4 mM of released sugar), compared to our hyperthermophilic designer cellulosome which exhibited 15- (after 24 h) to 25-fold (after 72 h) enhanced activity. The final level of cellulose-degrading activity of the trivalent hyperthermophilic designer cellulosome at 75 °C was superior to its performance at 60 °C and equivalent to that of the *Cl. thermocellum*-based system at its preferred temperature.

## Discussion

Interest in understanding efficient biomass deconstruction by cellulosomal systems continues to grow, from the perspective of both the basic and applied sciences. As a consequence, studies on artificial designer cellulosomes offer valuable tools for unraveling the mode of action and synergy of the complexed cellulosomal enzymes as well as guidelines for design of more efficient and more stable complexes. Designer cellulosome systems, containing both conventional and unconventional catalytic components [33, 70–74] have been demonstrated to improve synergistic action among the enzymes and consequent cellulosic biomass degradation. A key parameter for improving designer cellulosome technologies in the future could involve the thermostability of the constituents, whereby the use of hyperthermophilic enzymes would potentially provide distinct advantages, with respect to cost, handling ability, and efficiency [75, 76].

In a previous study [32], we reported the use of hyperthermostable enzymes, with a scaffoldin partially composed of cohesins derived from mesophilic bacteria. The presence of such mesophilic components appeared to limit the stability of the complex at temperatures that exceeded 60 °C. A subsequent study [33] described a more thermostable designer cellulosome, which employed three cohesin–dockerin pairs, originating from the most thermophilic organisms yet known to produce such protein modules [i.e., *A. fulgidus, Cl. thermocellum, Cl. clariflavum*]. In order to achieve enhanced performance at elevated temperatures, we selected for the latter study *Cl. thermocellum* enzymes that had been engineered to exhibit increased thermostability [77–79]. Indeed, the combination of thermostable enzymes and thermostabilized scaffoldin yielded designer cellulosomes that exhibited significant enhancement in cellulose digestion, compared to that of conventional designer cellulosomes comprising the respective wild-type enzymes. However, at temperatures above 65 °C, the thermostability and consequent performance of the complex were significantly reduced.

In the present work, we attempted to establish a highly thermostable and functional system that would deconstruct cellulosic substrates at extreme temperatures. For this purpose, we chose to combine the previously developed thermostabilized scaffoldin [33] with enzymes originating from the hyperthermophile *Ca. bescii.* In doing so, we succeeded to convert three hyperthermophilic enzymes into the cellulosomal mode by attaching a dockerin module without affecting significantly their functionality. We demonstrated increased levels of stability compared to those of the free components, whereby all components were combined into a single complex. Nevertheless, the resultant hyperthermostable cellulosome was not observed to exhibit increased performance over that of the free enzyme system.

Previous studies have demonstrated that the Cel9/48A enzyme is the most abundant and efficient cellulase in *Ca. bescii* [43, 65]. This bifunctional enzyme consists of three CBMs and two catalytic domains (GH9 and GH48), which possess both endo-processive and exoglucanase activities, respectively. Cel9/48A has been reported to outperform mixtures of commercially relevant exo- and endoglucanases on Avicel [43, 46]. Cel9/48A belongs to the multifunctional enzymatic paradigm, whereby highly active cellulose digestion results from combining complementary modules separated by long linkers. A synergistic mechanism has been proposed whereby the latter paradigm can be combined together with the other free enzyme or cellulosome paradigms [41, 80, 81]. Indeed, in nature, some cellulosome-producing bacteria clearly employ multiple approaches, including all three paradigms in the cell-free or cell-anchored modes [53, 82–85].

The expression of a full-length dockerin-bearing bifunctional Cel9/48A enzyme in *E. coli* proved challenging. The amount of protein produced was extremely low and required large culture volumes to obtain very small amounts of material (around 0.1 mg). Moreover, the isolated product exhibited numerous protein bands in the denaturing PAGE, indicating high levels of degradation. Since we were unable to produce reasonable quantities of the full-length dockerin-containing Cel9/48A, we decided to prepare individual forms of the two catalytic modules for inclusion into hyperthermophilic designer cellulosomes. Several studies have previously reported retention of activities following separation of catalytic modules derived from bifunctional enzymes [67, 81]. By splitting the Cel9/48A enzyme in two, we could convert each part into the cellulosomal mode and conserve the benefit of the proximity effect of the two catalytic modules by virtue of their presence within the same scaffoldin. Since the scaffoldin provides a CBM, the two CBMs from the parent Cel9/48A enzymes were excluded from the complex. The trivalent scaffoldin also allows integration of an additional enzyme into the complex. The architectural arrangement of the Cel9/48A and Cel5D enzymes on the scaffoldin was designed according to a previous study conducted with similar enzymes of the same families, derived from *T. fusca* and *Cl. thermocellum*, which demonstrated that positioning of GH5 and GH9 apart from each other has a direct positive effect on the enzymatic activity of the complex [34].

The role of linker in glycoside hydrolase activity has been investigated in previous works. Ruiz et al. [86] reported that linker length could be critical to the catalysis of *Bacillus subtilis* GH5’s. In another study, Tang et al. [87] demonstrated that flexible linker residues could enhance the catalytic efficiency of an endoglucanase from the fungus *Rhizopus stolonifera* var. *reflexus* TP-02. In contrast, Caspi et al. [88] found that the length of the linker between the catalytic module and the dockerin of *T. fusca* Cel5A’s had little, if any, effect on activity. The role of long repetitive linkers of the wild-type Cel9/48A and their contribution to enzyme activity remain unclear. In the present study, incorporation of a long linker connecting the GH9 and dockerin modules provided the designer cellulosome with a measurable increase in activity, whereas no improvement was detected with complexes that contained the GH48 with a long linker. However, unlike the native enzyme [42], the linkers in the recombinant forms are non-glycosylated, and their glycosylation could also affect overall activity of the enzyme itself and of the complex in general.

Although synergistic effects were not observed in this work for the hyperthermostable designer cellulosome over that of the free enzyme system, expansion of the numbers and types of enzymes could promote enhanced activities on native substrates, such as wheat straw. The increased activity at 75 °C of the hyperthermostable cellulosome over those of the native *Cl. thermocellum* cellulosome or previously prepared designer cellulosome of the same enzyme content [34] would indicate that the latter are not sufficiently stable over time at extreme temperatures.

Enzyme recycling in lignocellulosic biorefineries is a widely investigated, cost-effective process that has, however, been applied in very few industrial settings [89]. While most recycling technologies described in the literature encounter issues that can be attributed to the mesostable thermal characteristics of the enzymes, our system described herein would not require extensive temperature control, since the entire complex is thermostable. In this context, thermostable enzymes are known to be more stable in time, thus ensuring reutilization of the enzymes after recycling. Moreover, our system could allow enzyme recycling, conducted over 60 °C, without concern for enzyme denaturation. Another promising approach would entail recycling enzymes through contact with fresh cellulosic substrate. The enzymes as well as scaffoldin could be recycled in the form of the intact complex, thus avoiding enzyme loss and providing an efficient recycling system. The hyperthermostable system described in this work could thus improve the feasibility of recycling and could be integrated to other industries that deal, for example, with contaminated water treatment, clearing clogged pipes, or bio-mining [90]. The latter industrial processes would be facilitated by the use of thermostable enzymes and/or complexes thereof.

The limit of current hyperthermostable designer cellulosome systems is presumably contingent on and limited by the stability of the different cohesin–dockerin interactions and other scaffoldin components. It seems that Nature has not required the evolution of such strong stable interactions for development of specific cohesin–dockerin pairs that would act at extreme temperatures for long durations. We are currently restricted in our library of thermostable components to the type I and type II interactions confined to a short list of thermophilic microbial species. These include the archaeon, *A. fulgidus*, and selected thermophilic clostridia, e.g., *Cl. thermocellum, Cl. clariflavum, Cl. straminosolvens,* and their close relatives. Future expansion in the availability of hyperthermostable components, which can be used to integrate and maintain hyperthermostable enzymes into a defined complex at extreme temperatures, will be decisive in our continued attempts to conquer the barriers imposed by the recalcitrance of plant cell wall biomass.

## Conclusions

Creation of cost efficient and highly active designer cellulosomes that could be used at extreme temperatures for extended time periods would be a desirable endeavor. We have demonstrated herein that we can produce a highly active hyperthermostable designer cellulosome, which functions effectively at 75 °C for at least 72 h. The approach described in this communication offers a thermostable platform that could be used for other industrial purposes requiring high temperatures. Future work should focus on improving enzymatic synergy by further examining enzyme position and composition and extending the scaffoldin with additional thermostable cohesin–dockerin pairs, either through discovery of new, naturally occurring pairs or by conversion of mesophilic ones through directed evolution and/or genetic engineering.

## Additional files

**Additional file 1: Table S1.** Primers used in this work.

**Additional file 2: Figure S1.** Denaturing 12 % SDS-PAGE analysis of mutant proteins used in this study. Upper case characters (*G, T* and *V*) indicate the source of the cohesin modules and lower case (*g, t* and *v*) indicate the source of the dockerin module as given in Figure 1, where *G, g* refers to *Archaeoglobus fulgidus*, *T, t* refers to *Clostridium thermocellum*; and *V, v,* to *Clostridium clariflavum*.

**Additional file 3: Figure S2.** ELISA-based assay to confirm (A) the dockerin-binding functionality of the chimaeric enzyme and (B) the cohesin-binding specificity of Scaf*GTV*. In A, plates were coated with the target dockerin-bearing enzyme, and CBM-Cohesin was then introduced. In B, plates were coated with the scaffoldin, and xylanase fused to the indicated dockerins was then allowed to interact with the cohesins of Scaf*GTV.* Primary anti-rabbit anti-CBM and anti-xylanase, respectively, were then applied, followed by secondary goat anti-rabbit antibody conjugated to an HRP (horseradish peroxidase) [60]. The procedure of Barak, et al. [60] was followed for these experiments.

**Additional file 4: Figure S3.** Non-denaturing PAGE assay revealing the proper stoichiometric ratio of interaction between the chimaeric enzyme and its specific monovalent scaffoldin. Titration of the dockerin-bearing GH5-*t* with the Scaf*T* using the estimated molar ratios of components, yielded precise experimental data that indicated that the functional stoichiometric ratio was effectively between 1.1 and 1.2 GH5-*t*: Scaf*T* The functional ratios of the other components of the system were determined in like fashion.

**Additional file 5: Figure S4.** Thermostability of the Scaf*GTV*, complexed to the dockerin-bearing GH5 enzyme chimaeras (GH5-*g*, -*t* and -*v*). Thermostability was determined by non-denaturing PAGE after 0, 2, 6, 16 and 24 h of incubation at 70 °C, 75 °C and 80 °C.

**Additional file 6: Figure S5.** Activity of free enzymes and enzymes complexed to monovalent CBM-Coh scaffoldins. The dockerin-bearing *Ca. bescii* Cel9/48A derivatives contained long (*lk*) or short intermodular linkers. Activity was tested on microcrystalline cellulose (Avicel) substrate for 16 h at 75 °C.

## Abbreviations

CBM: carbohydrate-binding module
*Ca*: *Caldicellulosiruptor*
*Cl*: *Clostridium*
Coh: cohesin
DNS: dinitrosalicylic acid
Scaf: scaffoldin
GH: glycoside hydrolase
