# Supplementary material for: Creation of a functional hyperthermostable designer cellulosome
Source: Biotechnol Biofuels. 2019 Feb 28;12:44. doi: 10.1186/s13068-019-1386-y (PMC6394049; doi:10.1186/s13068-019-1386-y)
Supplement: Supplementary file 1 — Additional file 1: Table S1. Primers used in this work. [file 13068_2019_1386_MOESM1_ESM.docx]

Table S1. Primers used in this work.

| **Chimaeric enzymes** | **Cloning product** | **Forward primer 5’-3’** | **Reverse primer 5’-3’** | **Source of template** |
| --- | --- | --- | --- | --- |
| GH5 | Endoglucanase GH5 (without SLH and CBM) | atttatCC*ATG*Gcacagagcatactgtatgaaaagg | aattatGGTACCgc**gctattttcaaaattgatatcctc** | *C. bescii* DSM 6725 |
| GH5-*g* | Endoglucanase GH5- dockerin *g* | atatatGGTACC**agaagaagcaaacaagggagatgtg** | tattatCTCGAG**cttacccagtaagccattgcc** | 8A-*g^a^* |
| GH5-*t* | Endoglucanase GH5- dockerin *t* | atttatGGTACCg**acatataaagtacctggtactc** | aattatCTCGAG**gttcttgtacggcaatgtatc** | Cel48S^a^ |
| GH5-*v* | Endoglucanase GH5- dockerin *v* | atttatGGTACCg**tctcataaatttatctatggtgatg** | aattatCTCGAG**ttgttcttcaactgggaataagg** | BglA-*v^a^* |

UPPERCASE LETTERS: restriction site of endonucleases. **Bold fonts**: sequence complementary to the PCR template.

*Italic fonts:* start codon. All the cloning was conducted in pet28a plasmids.

*^a^* Moraïs, S., Stern, J., Kahn, A., Galanopoulou, A. P., Yoav, S., Shamshoum, M., Smith, M. A., Hatzinikolaou, D. G., Arnold, F. H., and Bayer, E. A. (2016) Enhancement of cellulosome-mediated deconstruction of cellulose by improving enzyme thermostability. *Biotechnol. Biofuels* *9:164*.
