# Supplementary material for: Creation of a functional hyperthermostable designer cellulosome
Source: Biotechnol Biofuels. 2019 Feb 28;12:44. doi: 10.1186/s13068-019-1386-y (PMC6394049; doi:10.1186/s13068-019-1386-y)
Supplement: Supplementary file 2 — Additional file 2: Figure S1. Denaturing 12 % SDS-PAGE analysis of mutant proteins used in this study. Upper case characters (G, T and V) indicate the source of the cohesin modules and lower case (g, t and v) indicate the source of the dockerin module as given in Figure 1, where G, g refers to Archaeoglobus fulgidus, T, t refers to Clostridium thermocellum; and V, v, to Clostridium clariflavum. [file 13068_2019_1386_MOESM2_ESM.docx]

**Figure S1.** Denaturing 12 % SDS-PAGE analysis of mutant proteins used in this study. Upper case characters (*G, T* and *V*) indicate the source of the cohesin modules and lower case (*g, t* and *v*) indicate the source of the dockerin module as given in Figure 1, where *G,g* refers to *Archaeoglobus fulgidus*, *T,t* refers to *Clostridium thermocellum*; and *V,v*, to *Clostridium clariflavum*.
