# Supplementary material for: Creation of a functional hyperthermostable designer cellulosome
Source: Biotechnol Biofuels. 2019 Feb 28;12:44. doi: 10.1186/s13068-019-1386-y (PMC6394049; doi:10.1186/s13068-019-1386-y)
Supplement: Supplementary file 3 — Additional file 3: Figure S2. ELISA-based assay to confirm (A) the dockerin-binding functionality of the chimaeric enzyme and (B) the cohesin-binding specificity of ScafGTV. In A, plates were coated with the target dockerin-bearing enzyme, and CBM-Cohesin was then introduced. In B, plates were coated with the scaffoldin, and xylanase fused to the indicated dockerins was then allowed to interact with the cohesins of ScafGTV. Primary anti-rabbit anti-CBM and anti-xylanase, respectively, were then applied, followed by secondary goat anti-rabbit antibody conjugated to an HRP (horseradish peroxidase) [60]. The procedure of Barak, et al. [60] was followed for these experiments. [file 13068_2019_1386_MOESM3_ESM.docx]

**Figure S2.** ELISA-based assay to confirm (A) the dockerin-binding functionality of the chimaeric enzyme and (B) the cohesin-binding specificity of Scaf*GTV*. In A, plates were coated with the target dockerin-bearing enzyme, and CBM-Cohesin was then introduced. In B, plates were coated with the scaffoldin, and xylanase fused to the indicated dockerins was then allowed to interact with the cohesins of Scaf*GTV.* Primary anti-rabbit anti-CBM and anti-xylanase, respectively, were then applied, followed by secondary goat anti-rabbit antibody conjugated to an HRP (horseradish peroxidase)^91^. The procedure of Barak, et al. (2005) was followed for these experiments.
