# Supplementary material for: Creation of a functional hyperthermostable designer cellulosome
Source: Biotechnol Biofuels. 2019 Feb 28;12:44. doi: 10.1186/s13068-019-1386-y (PMC6394049; doi:10.1186/s13068-019-1386-y)
Supplement: Supplementary file 4 — Additional file 4: Figure S3. Non-denaturing PAGE assay revealing the proper stoichiometric ratio of interaction between the chimaeric enzyme and its specific monovalent scaffoldin. Titration of the dockerin-bearing GH5-t with the ScafT using the estimated molar ratios of components, yielded precise experimental data that indicated that the functional stoichiometric ratio was effectively between 1.1 and 1.2 GH5-t: ScafT The functional ratios of the other components of the system were determined in like fashion. [file 13068_2019_1386_MOESM4_ESM.docx]

**Figure S3**. Non-denaturing PAGE assay revealing the proper stoichiometric ratio of interaction between the chimaeric enzyme and its specific monovalent scaffoldin. Titration of the dockerin-bearing GH5-*t* with the Scaf*T* using the estimated molar ratios of components, yielded precise experimental data that indicated that the functional stoichiometric ratio was effectively between 1.1 and 1.2 GH5-*t* : Scaf*T* The functional ratios of the other components of the system were determined in like fashion.
