# Supplementary material for: Creation of a functional hyperthermostable designer cellulosome
Source: Biotechnol Biofuels. 2019 Feb 28;12:44. doi: 10.1186/s13068-019-1386-y (PMC6394049; doi:10.1186/s13068-019-1386-y)
Supplement: Supplementary file 5 — Additional file 5: Figure S4. Thermostability of the ScafGTV, complexed to the dockerin-bearing GH5 enzyme chimaeras (GH5-g, -t and -v). Thermostability was determined by non-denaturing PAGE after 0, 2, 6, 16 and 24 h of incubation at 70 °C, 75 °C and 80 °C. [file 13068_2019_1386_MOESM5_ESM.docx]

**Figure S4**. Thermostability of Scaf*GTV*, complexed to the dockerin-bearing Cel5D enzyme chimaeras (GH5-*g*, -*t* and -*v*). Thermostability was determined by non-denaturing PAGE after 0, 2, 6, 16 and 24 h of incubation at 70°C, 75°C and 80°C.
