# Supplementary material for: Creation of a functional hyperthermostable designer cellulosome
Source: Biotechnol Biofuels. 2019 Feb 28;12:44. doi: 10.1186/s13068-019-1386-y (PMC6394049; doi:10.1186/s13068-019-1386-y)
Supplement: Supplementary file 6 — Additional file 6: Figure S5. Activity of free enzymes and enzymes complexed to monovalent CBM-Coh scaffoldins. The dockerin-bearing Ca. bescii Cel9/48A derivatives contained long (lk) or short intermodular linkers. Activity was tested on microcrystalline cellulose (Avicel) substrate for 16 h at 75 °C. [file 13068_2019_1386_MOESM6_ESM.docx]

**Figure S5.** Activity of free enzymes and enzymes complexed to monovalent CBM-Coh scaffoldins. The dockerin-bearing *Ca. bescii* Cel9/48A derivatives contained long (*lk*) or short intermodular linkers. Activity was tested on microcrystalline cellulose (Avicel) substrate for 16 h at 75°C.
